# Diversity in Genomic Studies: A Roadmap to Address the Imbalance

**DOI:** 10.1038/s41591-021-01672-4

**Published:** 2022-02-10

**Authors:** Segun Fatumo, Tinashe Chikowore, Ananyo Choudhury, Muhammad Ayub, Alicia R. Martin, Karoline Kuchenbäcker

**Affiliations:** 1 *The African Computational Genomics* (TACG) Research Group, MRC/UVRI and LSHTM, Entebbe, Uganda; 2 The Department of Non-communicable Disease Epidemiology, London School of Hygiene and Tropical Medicine, UK; 3 Sydney Brenner Institute for Molecular Bioscience, Faculty of Health Sciences, University of the Witwatersrand, Johannesburg, South Africa; 4 MRC/Wits Developmental Pathways for Health Research Unit, Department of Paediatrics, Faculty of Health Sciences, University of the Witwatersrand, Johannesburg, South Africa; 5 Division of Psychiatry, University College of London, London W1T 7NF, UK; 6 Analytic and Translational Genetics Unit, Massachusetts General Hospital, Boston, MA, USA; 7 Program in Medical and Population Genetics, Broad Institute of Harvard and MIT, Cambridge, MA, USA; 8 UCL Genetics Institute, University College London, London WC1E 6BT, UK

## Abstract

Two decades ago, the sequence of the first human genome was published. Since then, advances in genome technologies have resulted in whole genome sequencing and microarray-based genotyping of millions of human genomes. However, genetic and genomic studies are predominantly based on populations of European ancestry. This implies that the benefits of genomic research, including improving clinical care, understanding disease aetiology, early detection of diseases, better diagnosis, and rational drug design, may elude those underrepresented populations. Here, we describe factors that have contributed to the imbalance in representation of different populations. Leveraging our experiences in setting up genomic studies in diverse global populations, we propose a roadmap to enhancing inclusion and ensuring equal health benefits of genomics advances. This proposal highlights the importance of sincere concerted global efforts towards genomic equity to achieve the benefits of genomic medicine to all.

As of June 2021, the vast majority of genomics studies including genome-wide association studies (GWAS) have been conducted in individuals of European descent (86.3%), followed by East Asian (5.9%), African (1.1%), South Asian (0.8%), and Hispanic/Latino (0.08%) populations (Figure 1). While the proportion of samples from individuals of European ancestry has increased from 81% in 2016 to 86% in 2021, the proportion of samples from the underrepresented populations have either stagnated or decreased. Only genetic studies including participants with multiple ancestries have slightly increased to 4.8%. This shows that the diversifying progress has been slow. The genomic research community tends to extensively use resources with relatively straightforward access models, such UK Biobank which includes participants of mostly European descent, while other ancestry groups tend to have very few of such resources with limited access models. Data from the International HundredK+ Cohorts Consortium (IHCC), a recently established consortium of international cohort studies, also shows ancestral disparity (Figure 2).

Most of the data from non-European populations captured in the GWAS catalog and current genomic studies are individuals in diaspora populations. For example, the 1.1% of participants of African ancestry in the GWAS Catalog are mainly African Americans. The proportion of continental Africans in genomic studies is insignificant with respect to the prevailing genomic research. While there are five major African ethnolinguistic divisions, the African diaspora in the UK and USA predominantly consists of just one of these divisions, the Niger-Congo speakers^2^. Africans harbour a far greater amount of genetic and linguistic diversity (e.g., over 3000 indigenous languages) compared to populations from other continents^3,4^ and this diversity is largely partitioned by geography. However, more than 90% of these ethnolinguistic groups have no representative genetic data to date. Studying a small number African diaspora populations (African American and Black participants in the UK and Europe) and grouping all participants into a broad category of African ancestry will continue to promote imbalance, widen health disparities, and fail to capture the genetic diversity in Africa. Moreover, large-scale differences in environment and lifestyle could further limit the transferability of genetic insights (such as Polygenic Risk Score models) gained from diaspora populations to continental African populations^5^. This calls for immediate measures to address the genomic studies imbalance.

Here, we analyse what factors have contributed to the current inequalities in genomic studies. We highlight a few successful genomic studies in Africa, Asia, and Australia. We also reflect on the challenges and opportunities in setting up genomic studies in low-income countries. Based on our experience, we chart a roadmap (Figure 3) to increase diversity of populations in genomic studies which requires a concerted global effort. We emphasize that any successful roadmap must leverage established research infrastructure (e.g., existing cohorts), capacity, expertise, and leadership within local institutions in those countries.

## 2.0 Clinical unmet needs and genetic diversity imbalances in genomics

There are major scientific consequences and missed opportunities driven by Eurocentric study biases, including identifying novel associations with population-enriched variants, pinpointing causal variants for functional follow-up, improving genetic risk prediction accuracy for all populations (particularly underrepresented populations), and understanding shared versus unique genetic and environmental population risk factors that influence health outcomes^6–9^.

There are characteristics of the left-out populations that would benefit the international efforts of discovery of disease-causing variants. For example, African populations have the most genetic diversity followed by South Asians. This helps fine-map GWAS signals and identify target genes, an essential step in gaining mechanistic insights. These populations also have the most loss-of-function variants, which can aid interpretation of genomic function and understanding mutational constraints^10^. Endogamy within subgroups and consanguinity in some South Asian populations can enhance the power for discovery of recessive inheritance.

There are already clear examples of population-enriched clinically important variants only discovered in underrepresented populations; a few of these include associations between *APOL1* and chronic kidney disease^11^, variants in *G6PD* that contribute to missed diabetes diagnosis^12^, and loss-of-function variants in *PCSK9* that lower LDL cholesterol (the discovery that led to *PCSK9* inhibitor drugs)^13^, all of which were identified in populations with African ancestry.

Additionally, polygenic risk scores have become increasingly predictive as GWAS have grown and increased in power. Interest in their predictive utility, which is now comparable to other biomarkers commonly used for screening actionable diseases areas such as breast cancer and cardiovascular disease^14,15^, has raised their potential for clinical translation alongside other risk factors^16^. However, their accuracy decays with increasing genetic distance from the study cohort^17,18^; a previous study showed that Eurocentric GWAS results for several traits produce PRS that are 2-fold and 4.5-fold more accuracy in individuals with European than East Asian and African ancestry, respectively^6^. Thus, increasing diversity in genomics is critical to ensure that translation improves health outcomes for all and does not exacerbate health disparities^6^.

Imbalanced ancestral diversity also pervades data sets with whole genome and whole exome sequencing. This is of particular concern for resources that are available as reference panels for genotype imputation. For example, the most widely used genomic reference panel consisting of the 1000 Genomes Project dataset, has been shown to represent a minority of ancestry groups found in mainland South Asia and Africa^19^. This limits the post-imputation coverage of genomic variation for many populations.

## 3.0 Factors contributing to the current inequalities in genomic studies

The dominance of European and American scientists in genomic research stems from advances in genomic technologies, infrastructure, and the better funding opportunities. These are a consequence of structural advantages, some of which are related to historical and present-day exploitation. The lack of diversity in researchers is a crucial driver of bias in genetic studies^20^. Previous work shows that investigators have personal connections to their countries of origin, leading to their prioritization in research^21^.

The exclusion of data from individuals of non-European descent, including those living in European and North American countries, has been justified by concerns about population stratification as well as lack of capacity and analytical expertise with respect to multi-ancestry cohorts. Advances in the development of genetic technologies that capture the variation in diverse populations coupled with requisite analytical tools now offer an opportunity to explore genomic studies in multi-ancestry populations.

Large-scale genetic studies are expensive and time-intensive, requiring continuity of expertise. Several countries have faced political instability that has made investments in genomic research erratic. Recent strategic funding by the NIH and Wellcome Trust through the Human Heredity and Health in Africa initiative has led to the birth of genome-wide association studies on the African continent.

Moreover, for participants to engage in research they need to trust the researchers. Past history of research abuse and exploitation has impacted on the ability of researchers to work with diverse communities^21^. The limited understanding of the genetic concepts among some indigenous populations and the paucity of data on effective models for community engagement may also contribute to poor enrollment of research participants in some population groups^22^. When membership on community advisory boards is sustained by members who meet with researchers, they may facilitate community engagement.

There are two broad groups of under-represented populations; residents of low and middle income countrie^23^ and indigenous and minority groups across the globe ^24^. The factors that have caused unequal representation are overlapping in two groups.

The burden of historical injustices including coercion and deception in research (Nazi Medical Experiments|Holocaust Encyclopedia (ushmm.org),^25,26^ and negative experience with the healthcare system^27^ results in lack of trust in research. The mutual suspicion and lack of trust is a significant cause for scientists to avoid enrolling indigenous groups and for indigenous groups to participate in research.

For low and middle income countries resources in terms of funds, institutional capacity and skilled work force are major barriers ^28^. Low and middle income countries have limited funds to invest on research and genomic research does not make its way on their list of priorities. For genomic research scientists in these countries depend on the funding from high income countries mostly through collaborative efforts. The funding agencies policies and priorities influence the decisions about the focus of research and they set the research agenda in these countries ^29,30^. In collaborative research scientists from low and middle income countries are under-represented as first and last authors and that impacts their motivation to engage in big collaborations ^31^.

Lack of expertise in ethical, legal, social implications relevant to genomics research has hindered the conduct of research and data sharing ^32,33^. Creating expertise in this area and making ELSI considerations integral part of the study design will address this gap. Local adaptation of the available guidance can help ^34^

## 4.0 What has worked in setting up genomic studies in underrepresented populations

Despite the unequal representation of ancestry groups in genomic research, some studies in underrepresented populations have been very successful. In this section, we discuss flourishing genomic studies in underrepresented populations, mostly from LMICs countries from Africa, Asia, Latin America and also show that the problem of genomic underrepresentation is not restricted to LMICs with a case study from Australia. We reflect on factors contributing to their successes.

### 4.1 Africa

Large-scale genomics research in Africa has so far been driven mainly by international funding, with very few examples of government funded national level initiatives such as the Southern African human genome programme ^35^. MalariaGen ^36^ was among the first studies to be based on a cohort that spanned multiple African countries. The focus of this study on the genetics of both the parasite and the host enabled it to capture snapshots of human genetic diversity, especially in some of the malaria endemic geographic regions of Africa. However, the H3Africa consortium was the first major pan-African study to have a comprehensive spread across the continent as well as a wide variety of diseases and traits ^37^. In addition to investigations of both communicable and non-communicable diseases the efforts of the consortium has contributed to developments in several major aspects of genetics research such as ethics and community engagement, design of a genotyping array, data sharing and governance, disease awareness, bioinformatics skills and analysis tools ^38^. In the following paragraphs we describe two cohorts, the Uganda genome resource study and the AWI-Gen study (a collaborative centre of the H3Africa consortium) that are population cross-sectional and have been generating key insights into genetics of a number of cardiometabolic traits and diseases.

#### 4.1.1 Strategic collaboration and capacity building: The Uganda Genome Resource

Genomic studies in countries in Africa provide unique opportunities to understand disease etiology, human diversity, and population history. The Uganda genome resource represents the largest published genome study of continental Africans to date^39^. This genomic study leveraged already existing strategic collaboration between the Uganda Virus Research Institute, University of Cambridge, and Sanger Institute, United Kingdom. In 1989, the Uganda General Population Cohort was established by the Uganda Virus Research Institute and partners to examine trends in prevalence and incidence of HIV infection and their determinants^40^. A genomic study of communicable and non-communicable diseases was launched in 2011 with this cohort. The successful implementation of genomic research here can be attributed to existing local infrastructure in Uganda, long-standing collaborations with genomic centres of excellence in the UK, and strategic funding that included a research capacity building component. For example, the author Segun Fatumo is a former H3Africa Bioinformatics Network (H3ABioNet) fellow who was funded to do postdoctoral research training in statistical genetics and bioinformatics at the Sanger Institute and University of Cambridge. During this training, he was strategically positioned to take a lead role in analyses of the Uganda genome resource. Following this training and research, Segun Fatumo has since continued to maintain the genomic resources locally and led other genomic studies^41–44^. Furthermore, this resource has enabled significant new insights for population genetics and genetic epidemiology. For example, a genetic variant known to cause ɑ thalassemia was significantly associated with glycated haemoglobin, an indicator commonly used in the diagnosis of diabetes^39^. This variant is thought to have become more frequent among African populations because it can prevent severe malaria^39^.

#### 4.1.2 Building on existing resources - Africa Wits-INDEPTH partnership for Genomic Studies (AWI-GEN)

AWI-Gen is an NIH funded cross-sectional population cohort of about 12,000 older adults (40-60 years) from 6 centres spanning 4 African countries - Ghana, Burkina Faso, Kenya, and South Africa. It was set up by a strategic regional partnership between the University of the Witwatersrand, Johannesburg and the International Network for the Demographic Evaluation of Populations and Their Health (INDEPTH) study. The existing Health and Demographic Surveillance System centres and the Developmental Pathways for Health Research Unit have longitudinal cohorts which provided the research infrastructure, including long-standing community engagement, trained fieldworkers, and detailed longitudinal demographic and phenotype data. This mutually beneficial partnership enabled the project to span Africa with a wide representation of social and genetic variability that has resulted in more than 40 publications. These span disciplines including epidemiology, disease awareness, population genetics, candidate gene studies, and gene environment interaction^45–49^. Several major GWASs are close to publication and have led to partnerships with large-global consortia such as Global Lipid Genetics Consortium and Cohorts for Heart and Aging Research in Genomic Epidemiology (CHARGE) study. Additional funding from these partnerships has enabled the transformation of AWI-Gen into a longitudinal cohort. The achievements of the AWI-Gen study are in part attributable to the strategy of building on existing resources and forming long-term partnerships based on benefit sharing among institutions within low and middle income countries (LMIC) settings.

A major achievement of these studies beyond research outputs was the spread of bioinformatics/genomics skills across the continent. For example the annual Introduction to Bioinformatics course run by the H3A-BioNet (Bioinformatics network of the H3A consortium) has trained over three thousand students in the last 8 years ^50^. In addition the network has hosted more than 30 workshops for basic and advanced training in areas such as GWAS, NGS, microbiome and data Similarly, the set up and development of several biobanks across the continent associated with these projects could have a catalytic effect for research and development initiatives in future. Finally, as these studies reach completion we anticipate that some of the outcomes would be able to benefit the communities and also contribute to the bio-economic landscape of the respective LMICs.

### 4.2 Asia

#### 4.2.1 The importance of funding: Pakistan Alliance on genetic RisK factors for Health (PARKH)

South Asians make up one sixth of the world population, with 1.38 billion people living in India alone. Pakistan and many other countries in the region have a high rate of consanguineous marriages and have been the focus of gene mapping studies for recessive disorders for the last few decades. There is a long list of disorders for which mutations have been discovered in families from these regions including hearing impairment ^51^, intellectual disability ^52^, microcephaly ^53^ and visual conditions ^54^. These studies have contributed to the global efforts for study of genetic causes of recessive disorders and their underlying biology. In the process genotyping and sequencing data has been created that can be leveraged to address questions about population structure, population specific allele frequencies and ancestry ^55^. This will require collaborative networks, data storage and access mechanisms that follow ELSI guidelines. The Greater Middle East (GME) Variome Project is one such successful example (GME (ucsd.edu).

However, South Asians are particularly underrepresented in genomic research of complex diseases. With a target recruitment of 30,000 psychiatric patients and 15,000 control participants, PARKH (Pakistan Alliance on genetic RisK factors for Health) is one of the largest international case control efforts with genetic data. Over a time span of 20 years, the team built up extensive links with other institutions across Pakistan through small family-based studies^52,56,57^, which eventually enabled a sizable pilot sample collection. Local connections, cultural understanding, knowledge of the administrative and regulatory processes, resilience, and the flexibility to navigate an ever-changing research landscape have been the key factors in the success of these projects. The collaboration between Pakistani, US- and UK-based researchers was a decisive factor in opening up access to funding resources. For example, one of the three PARK sister studies, DIVERGE, is funded by a Starting Grant from the European Research Council (€2.5 million) for which only researchers in the European Union and a select group of partner countries are eligible. The two other sister studies, the GENetics of SChizophRenia In Pakistan (GEN-SCRIP) (R01MH112904-01)and GENetics of BipoLar Disorder In Pakistan (GEN-BLIP) (R01MH12377) have been funded by the US National Institute of Mental Health (NIMH). PARKH demonstrates that building and maintaining infrastructure and a network for data collection as well as international collaborations can be the foundation for repeated funding success and may serve as motivation for ambitious strategies at large-scale. In the case of PARKH, none of the funders provided a dedicated capacity building component. Rather, the investigators implemented their own strategies that included hiring local researchers for diverse roles.

Study design can also play an important role in enabling sustained research activity. For the DIVERGE study, a dedicated cross-disciplinary working group designed a protocol that captures diverse outcomes and putative risk factors for depression to enable multidisciplinary research on depression genetics, pharmacogenetics, interactions between genes and traumatic life events, and epidemiological analyses of socioeconomic factors. Importantly, local investigators took key roles in the study design to ensure that factors relevant to the studied populations are captured in the data collection.

### 4.3 Latin America

#### The importance of consortium building to facilitate aggregation of large-scale genomic data - The Latin American Genomics Consortium

Latin American refers to a pan-ethnicity used for the large, diverse group of people who come from Latin American countries. Additionally, people in other countries who identify with Latin American origins are often identified as Hispanic or Latinx American. For example, “Latino” is the only official ethnic group in the USA. Latinx populations have complex ancestry including recent admixture. Commonly used analytical approaches may not sufficiently address population stratification in these groups. Principal components as covariates only account for global ancestry but not for local ancestry for a given genomic region. In addition to the lack of dedicated genomic studies in these groups, individuals with admixed ancestry are systematically excluded from existing studies due to these concerns of population stratification. The recently established Latin American Genomics Consortium aims to address this in the area of psychiatric genetics (https://latinamericangenomicsconsortium.org). This group includes over 100 scientists from eight Latin American countries, Puerto Rico and the USA. The group harmonises data from existing cohorts and has a total of 100,000 samples, mostly from the USA. They also plan to recruit new participants and establish a biobank. Developing methods for samples with admixed ancestry is an active field of research. A promising albeit computationally intensive approach is Tractor which identifies haplotype segments and assigns them to ancestral origins, followed by an ancestry-specific association analysis^58^.

### 4.4 Australia

#### 4.4 The importance of the community in setting research priorities - The Tiwi Island Aboriginal Population

Aboriginal and Torres Strait Islander people in Australia are one of the largest indigenous populations in the world. They comprise hundreds of groups, each with their own distinct language, history, and cultural traditions. The Tiwi people have proactively participated and engaged with research of chronic and kidney disease in their community for more than 30 years or more. The stakeholders in the community provide ethical guidance for researchers and support for communities^59^. The Tiwi Land Council signed an historic research agreement to formalize Tiwi control of the research priorities, research information, and samples including biobanking in genomic studies^60^. At one point, the Tiwi community raised local financial support and raised external funds, specifically the Stanley Tipiloura Fund to support research^61^. Members of the Tiwi community have worked as staff in all research projects conducted within their community^61^, including encouraging application of genetics research to determine their origins, migrations, customs, relationships, and health issues^61^. The Tiwi Island Aboriginal Population is an example of best practice for indigenous-led initiative with a substantial proportion of indigenous researchers and leaders. The recently launched National Centre for Indigenous Genomics (NCIG) is governed by an indigenous-majority board and demonstrates genuine partnerships with community and stakeholders.

The initial successes of these cohorts/studies described here illustrate that with sufficient funding it is possible for groups at LMIC institutions to scale up in resources and skills to be able to do high quality research in less than a decade’s span. These instances should motivate funders to support both ongoing and new ventures that are led by LMIC researchers. Moreover, publications in top tier journals and presentations in the major conferences have also provided them the opportunity to participate in/contribute to large-global studies. We hope that in future they would not only be able to extend their research to larger cohorts but would be able to move closer to leading at least some of the large-scale global studies. As an example of this, two authors from AWI-Gen study (including one of the authors of the current manuscript, Tinashe Chikoware) were recently provided the opportunity to co-lead one of the CHARGE consortium Phase 2 studies.

## 5.0 A Roadmap for establishing sustainable diverse genomics research worldwide

**Figure 3:**
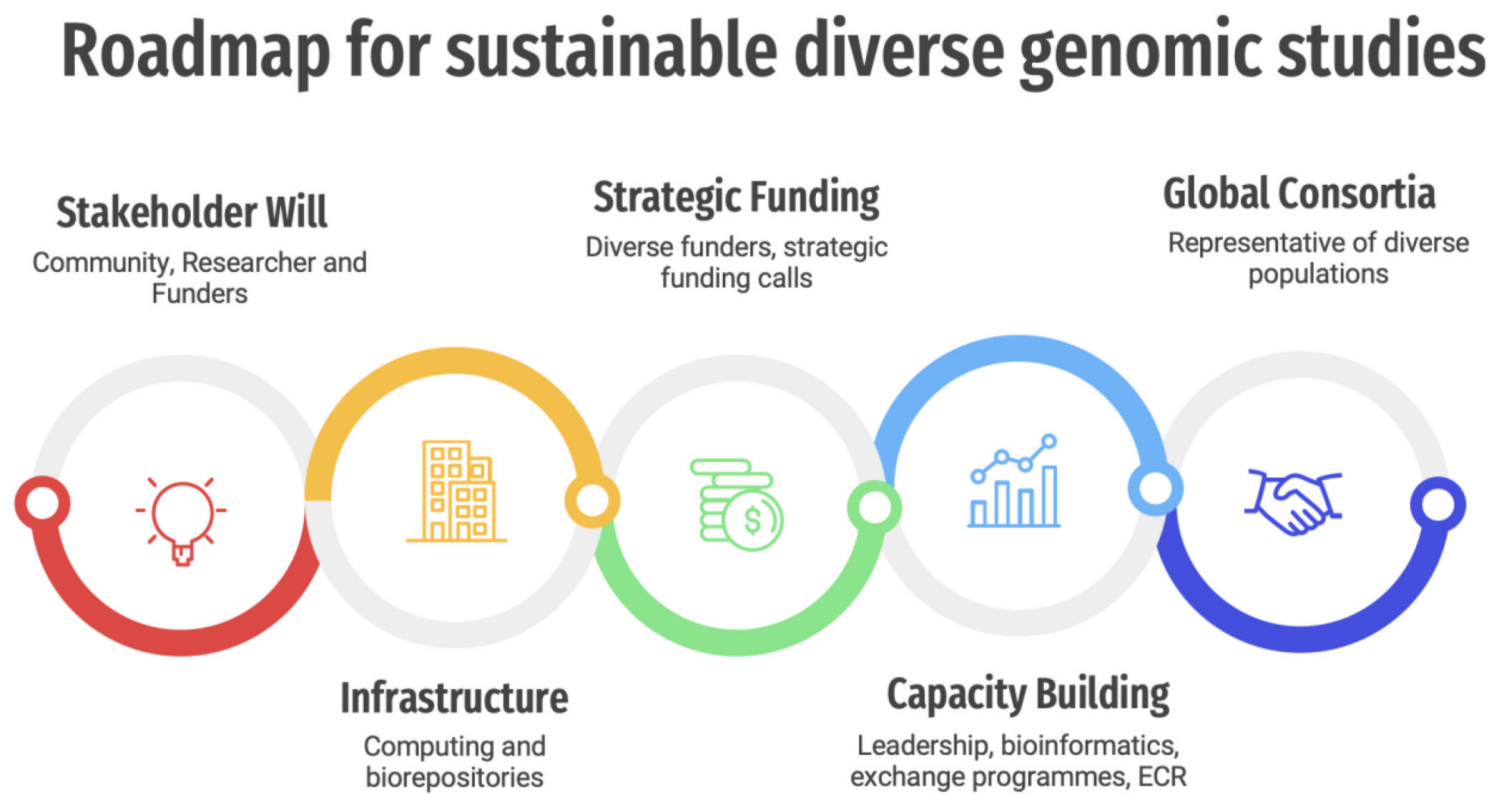
Roadmap showing the key pillars for setting up and sustaining diverse global genomic studies.

Given our experiences in setting up genomic studies in diverse populations, we recommend key priority steps illustrated in the roadmap infographic (Figure 3)

### 5.1 Stakeholder will

The importance of diversity in research studies has been known for a long time, as evidenced by legislation and guidelines for example enacted in the USA in 1993 to increase participation of women and minority groups in clinical studies [NIH Revitalization Act of 1993 Public Law 103-43. Federal Register, 59FR14508]. However, participation of the minority groups such as Hispanics and African-Americans has remained limited in America^20^. The lack of diversity in genomics requires boldness and willingness of the varied stakeholders, including research institutions, researchers, participants, funders, governments to collaboratively work together to address this imbalance. To help correct the lack of diversity in genomic research, there is need for:-

1. Research institutions to be willing to ensure they have a diverse workforce. This has been shown to improve trust among minority groups leading to improved recruitment. Diverse researchers have been reported to be more interested in studying about their population groups, thereby increasing diversity in genomics. Notably, programs such as the NIH UNITE have been set up to address structural racism in the workplace and ensure diverse researchers have equitable access to opportunities in biomedical research. In view of the global nature of research, there is need for institutions that support open access to research outputs which will help other research in the world to carry forward similar work and also replicated in diverse settings.
2. Researchers to be willing to form genuine partnerships with communities that result in ethical conduct of genetic research which benefits all^22^. It has been suggested that researchers need to take time to address the historical perceptions and distrust of clinical research by minority groups by taking time to help them understand the goals of the genomic studies and clarifying concerns of potential harm. Ultimately leading them to integrate participants values and expectations in the implementation of genomic studies^27^.
3. Research participants from minority groups to be willing to participate in genomic studies. It has been reported that when the participants trust the researchers and their governments, they are willing to participate and even offer broad consent in BioBank studies^62^. Thereby indicating that if the researchers and government work together to ensure ethical and trustworthy research is conducted more minority groups will participate in research. However, there is need for more research that will inform policy with regards to: who benefits from commercialisation of the research outputs; how genomic sovereignty can be maintained in the context of broad or tiered consent.
4. Funders to be willing to set up strategic funding schemes which promote research of underrepresented population groups. Genomic research in underrepresented population groups has been noted to require more time and resources and funders need to be able to commit to this. Most scientists from these population groups have a lower competitive edge compared to those of European ancestry and they will need earmarked funding for them to ensure they grow capacity to be able to compete for grants in the future. The Human Hereditary and Health in Africa (H3Africa) and the Data Science for Health Discovery and Innovation in Africa are examples of strategic funding that the NIH has committed to bolster genetic research in Africa.
5. Governments to institute policies that create environments conducive for sustainable diverse genomics studies. A number of governments are realising the potential and value of genomic studies even among underrepresented populations. Examples such as the China Kadoorie Biobank and the South African Human Genome Project offer a promise that if more governments take such steps sustainable diverse genomics may be realised.

Studies that are focused on cohorts from previously marginalized populations have the additional burden of managing the damage that has been caused by some of the earlier studies. The extractive attitude of some of the initial studies coupled with the lack of engagement with the community and the appreciation of their beliefs and sentiments has led to a general distrust in researchers among some of these communities. Therefore, in addition to an extensive and prolonged engagement with the community, studies focus on areas that are health priorities for the respective communities and have a potential to result in tangible benefits to them are essential to heal the scars and help these communities to view researchers as allies and partners.

### 5.2 Funding

Genetic research is expensive, making it a secondary priority for funding in LMIC. One route towards increasing studies in underrepresented global populations is by leveraging funding mechanisms from institutions in research intensive nations and international institutions. Funders have an opportunity to help address imbalances in global genetics research through their research priorities. Dedicated funding calls, such as the “Genetic Architecture of Mental Disorders in Ancestrally Diverse Populations” by the National Institute of Mental Health in the US, can be a strategic tool to empower fast progress.

#### Barriers to access

Many funding calls are exclusively targeted to researchers at institutions in the funder’s country. Given the immense general benefits of increasing diversity in genetic research, funders should reconsider such restrictions. In addition to eligibility restrictions, fewer researchers in LMICs have track records competitive for large funding calls due to the limited research capacity, infrastructure, and funding at their local institutions. This catch-22 makes it very difficult for those researchers to build up large genomic studies without collaborators from research intensive nations.

#### Collaboration

For most of the case studies we have presented here, collaborations between local investigators and those from research-intensive nations were critical for funding success. Collaborations can provide diverse expertise that includes competitive research track records, experience in grant writing, administrative support, and the necessary local expertise and knowledge about the target population. Therefore, networking and building long-lasting productive collaborations remains a key route for investigators to access funding for large-scale genetics research. However, power imbalance needs to be considered in collaborations with researchers from research intensive nations. When capacity building is part of it, this collaborative approach may eventually support local expertise sufficiently to enable more genomic research led by investigators in LMIC’s. Moreover, data sharing agreements are important to ensure the interests of the local researcher are respected. A final point to consider is potential negative reactions by some members of local communities to initiatives led by foreigners.

#### Sustainability

Sustainability should be a primary consideration for awarded funds to most effectively improve the diversity of genomic studies long-term. Many funding calls do not provide a dedicated capacity building component. In these cases, researchers can still invest funds to enhance local capacity for long-term benefits, such as by hiring local students or researchers for training or research positions (see also Capacity Building).

### 5.3 Infrastructure and administrative components

To conduct cohort based genomic research, it is not only critical to access some key infrastructure components but also important to align the study with the legal, administrative and ethical frameworks applicable at the institutional and national level. We have summarized in **S Table 1**, some of the pre-study administrative components that were taken into consideration by the cohorts that we have described above. A comprehensive understanding of ethical concerns, regulations and policies could enable researchers to avoid major delays in cross-border shipping of biological samples and also ensure the ability to re-use/share these valuable datasets in future. Most of the studies described above report pre-study consultation with legal experts (often available via their institutions) and implementation of necessary material and data transfer agreements to ensure efficient movement of samples and data. **S Table 2** lists the three major ways by which the core infrastructure involved in the actual genomic data capture and analysis was accessed by these cohorts. As evident from the table, the infrastructure for steps such as sample processing, biobanking, genotyping or sequencing and computational analysis are often outsourced or accessed via local and international collaborations (**S Table2**). However, developing the ability and infrastructure to be able to do one or more of these at the institutional level could be a major capital for securing continued funding for the study and future research.

### 5.4 Capacity Building

To narrow gaps in genomic studies for underrepresented populations, training and education models that retain trained individuals are critical; these provide knock-on opportunities to transfer technology and knowledge locally, thereby creating a critical mass of appropriately trained individuals.

For example, capacity development has been one of the key aims of the AWI-Gen study. In addition to training over 20 postgraduate students and postdoctoral fellows in statistical genetics, the consortium has been a key contributor to several major GWAS training initiatives on the continent. This includes hosting/organizing courses and workshops independently as well as in partnership with bodies such as H3Africa Bioinformatics Network, Wellcome Trust Overseas course, and Sweden South Africa University Forum. AWI-Gen has also been a key contributor to the development of the H3Africa GWAS pipeline and Imputation facility that is anticipated to help future genomics research on the continent. However, like most other studies in LMIC settings, retaining trained students and scientists continues to be a challenge that this study must deal with.

In Pakistan, PARKH team utilizes their international links to support researchers to visit labs in the US and Canada for training. These visits were organized in collaboration with the Higher Education Commission of Pakistan that guarantees that scholars return to work in their institutions. We have formed virtual analysis teams bringing together experts in the United States and Canada and trainees and junior faculty from Pakistan. Senior researchers from our collaborative network have co-supervised graduate students from Pakistani universities.

### 5.5 Partnership with Global Consortia

**I**ncreasing diversity in genomic studies contributes to more robust findings from replicated results as well as novel discoveries, particularly when combined with existing large-scale studies. Developing local research capacity enables contributions to global genomics consortia, as demonstrated in several consortia already such as the Global Lipids Genetics Consortium^63^, GIANT Consortium, Psychiatric Genomics Consortium and other major initiatives. These have dual and mutual benefits by enabling the discovery of ancestry-specific findings, raising the profile of these findings to a broader audience, and enhancing the careers of local contributing investigators. Participation in global consortia by diverse groups requires trust, which can only be built when all contributors benefit.

## Conclusion

Despite some notable efforts, representation of non-European ancestry groups in genetic research remains low. This particularly affects diverse global populations. The benefits of greater diversity extend beyond the studied population. We present a vision with a concrete roadmap on how to address this imbalance. Leveraging established local infrastructure and offering strategic funding that is tied to capacity building could empower sustainable global research. To be successful in achieving equitable inclusion of underrepresented groups in genomic studies, the stakeholders must stimulate local participation, build trust, and ensure mutual respect.

## Figures and Tables

**Figure 1:**
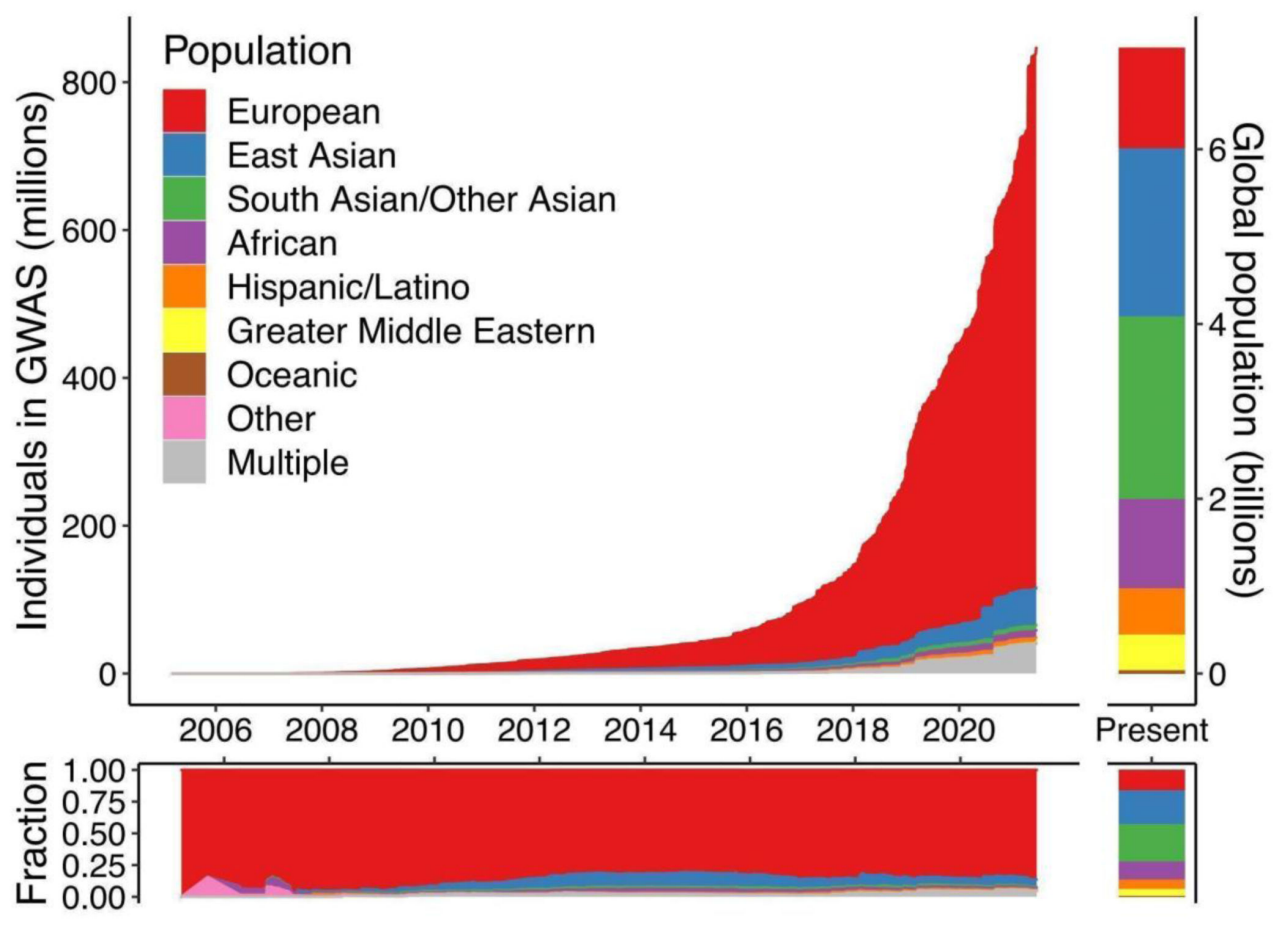
The proportion of samples from individuals cumulatively reported by GWAS Catalog^1^ as of July 8, 2021.

**Figure 2:**
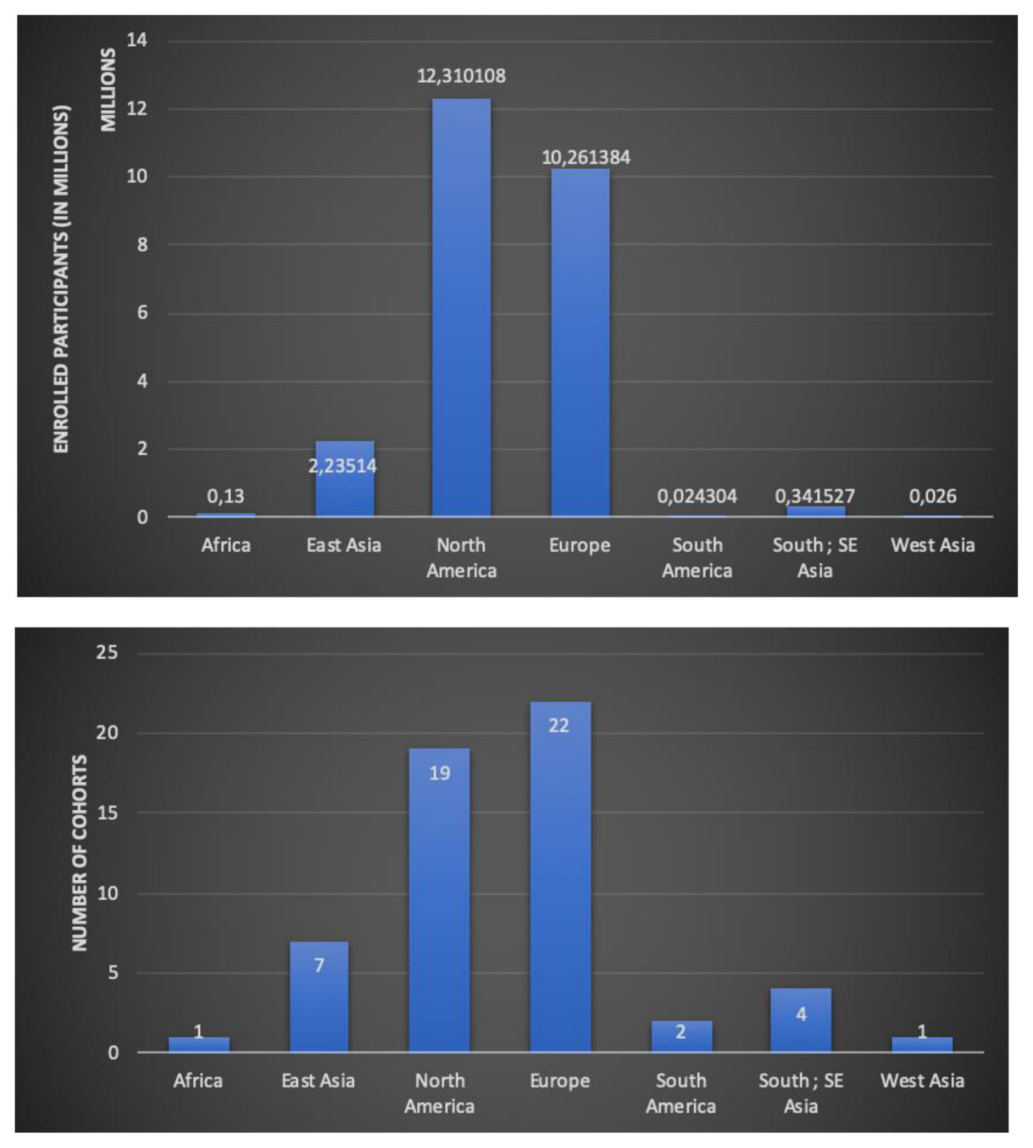
Disparity in representations of continents will increase in the next few years without immediate measures to increase diversity in genomic studies. Upcoming large-scale (>100 K participant) cohort-based studies included within the IHCC was employed as an indicator of the representation of various continents in genomics research over the next few years. (a) Number of enrolled participants from each geographic region (b) Number of cohorts from each geographic region. The estimates are based on cohorts that are collecting or aim to collect genomic data (https://ihccglobal.org/membercohorts/).
